# The impact of age-related increase in passive muscle stiffness on simulated upper limb reaching

**DOI:** 10.1098/rsos.221453

**Published:** 2023-02-08

**Authors:** Tiina Murtola, Christopher Richards

**Affiliations:** Department of Comparative Biomedical Sciences, Royal Veterinary College, London, UK

**Keywords:** ageing, manual aiming movements, passive stiffness, Hill-type muscle model

## Abstract

Ageing changes the musculoskeletal and neural systems, potentially affecting a person’s ability to perform daily living activities. One of these changes is increased passive stiffness of muscles, but its contribution to performance is difficult to separate experimentally from other ageing effects such as loss of muscle strength or cognitive function. A computational upper limb model was used to study the effects of increasing passive muscle stiffness on reaching performance across the model’s workspace (all points reachable with a given model geometry). The simulations indicated that increased muscle stiffness alone caused deterioration of reaching accuracy, starting from the edges of the workspace. Re-tuning the model’s control parameters to match the ageing muscle properties does not fully reverse ageing effects but can improve accuracy in selected regions of the workspace. The results suggest that age-related muscle stiffening, isolated from other ageing effects, impairs reaching performance. The model also exhibited oscillatory instability in a few simulations when the controller was tuned to the presence of passive muscle stiffness. This instability is not observed in humans, implying the presence of natural stabilizing strategies, thus pointing to the adaptive capacity of neural control systems as a potential area of future investigation in age-related muscle stiffening.

## 1. Introduction

Ageing affects the musculoskeletal system through changes in both neural and muscular function, resulting in alterations in an individual’s ability to perform daily living activities. Compared to young adults, older individuals have been observed to have, for example, increased risk of falling (e.g. [1,2]), altered gait patterns (e.g. [3,4]) and reduced upper limb functionality [5]. This deterioration in performance and behavioural outcomes has been linked with age-related changes in muscle properties, such as lower muscle volume [6–8], reduced maximal voluntary torques [6,9] and reduced maximal shortening velocity of muscles [8,9]. Understanding the full causal chains from histological changes to performance outcomes remains a challenge, however, due to the multifactorial nature of ageing and the difficulty of studying each factor in isolation in experimental settings. This is particularly true for upper limb tasks which do not require maximal strength or speed, and hence their limiting factors are not necessarily straightforward to measure. Yet failures in reaching tasks, and subsequent potential loss of balance, are a contributing factor for falls in the elderly [2].

Prior work has identified multiple age-related factors underlying changes in muscle function, such as the reduced number of alpha motor neurons [10], changes in the number and cross-sectional area of different muscle fibre types and hence their contribution to muscle contractions [8,11] and increasing proportion of non-contractile tissue within muscles [12,13]. In the present study, we focus on the age-related increase in the passive stiffness of muscles which has been proposed to arise mainly from changes in intramuscular connective tissue (IMCT) [14,15], although other sources for age-related muscle stiffening, such as changes in muscle fibre properties, may also exist [16]. In general, the IMCT-related muscle stiffening stems from two age-related effects. Firstly, the amount of IMCT increases with age; specifically, the fraction of cross-sectional area increases [12,15], as do the absolute and relative volumes [13]. Secondly, connective tissue has been observed to become intrinsically stiffer with age [14,15,17]. Since IMCT stretches in parallel with muscle fibres during muscle elongation, either increasing its amount or intrinsic stiffness results in the higher passive stiffness observed at the whole-muscle level [12,14,15]. Therefore, for simplicity, the current study addresses the effects of increasing whole-muscle passive stiffness, regardless of the physiological origin of the stiffening itself, as this aligns with the general observations from both humans [7,13,15,16] and rodents [12,14,17,18].

The causal link between changes in muscle properties and behaviour is difficult to establish *in vivo* due to the invasive nature of muscle physiological experiments. Moreover, *in vivo* approaches cannot easily isolate the influence of connective tissue stiffening from other ageing effects within the body systems (see [19] for review). Biomechanical modelling circumvents these problems by offering tools for studying changes in the musculoskeletal system separately or in controllable combinations. In particular, simplified models offer tractability which enables identifying causal relationships. Modelling has previously been used to investigate the effects of ageing muscles (achieved via parameter adjustments) on ankle joint torques [20,21] and the effects of motorneuron and motor unit changes on the produced muscle force in the elderly [22,23]. The effects of passive stiffness increases have, however, previously either been intentionally excluded [21] or compounded with multiple other parameter changes [20].

The aim of the present study is to use a simple biomechanical upper limb model [24] to investigate how reaching characteristics are affected when the passive muscle stiffness increases. The movement of the model arm is controlled via antagonistic Hill-type muscle pairs with realistic excitation-to-activation dynamics [25]. The muscle excitations for the reaching movements are computed by a PD controller representing neural motor control. As discussed above, previous studies suggest that there are multiple potential ways in which the stiffness characteristics of muscles may change with ageing, each most naturally represented by different modifications to the muscle model. Thus, we exploit the versatility of computational modelling to investigate the impact of different pathways to increased stiffness. Our simulations test two main hypotheses. Firstly, we predict that increased muscle stiffness causes a decline in reaching performance. Specifically, we expect age-related reaching failures to depend on the target position within the model’s workspace (i.e. the set of all points the model can reach given its geometry). In particular, the targets near the edges of the workspace require certain joints to reach extreme angles, causing maximal muscle elongation which is more likely to stretch passive tissues. Furthermore, even in the absence of passive stiffness, prior work suggests that a straightened arm is less controllable [24], which may compound the stiffening effects. Secondly, we predict that age-related changes can be compensated for by adjusting the parameters of the neuromuscular controller, as is the case with changes in other intrinsic muscle properties [24].

## 2. Methods

### 2.1. The upper limb model

The two-dimensional upper limb model of Murtola & Richards [24], consisting of four segments (stationary upper chest, and moving upper arm, forearm and hand) and three range-limited hinge joints, is used. This model performs reaching movements in the horizontal plane and is controlled via excitation of six Hill-type muscles (one flexor-extensor^1^ pair per joint), whose force generation depends on their force–length and force–velocity characteristics as well as on third-order activation dynamics (following [25]) which converts a muscle excitation signal into the activation state of the muscle. Muscle excitations are computed by a predictive PD controller under the assumption of no co-excitation (i.e. the flexor and extensor of a joint are not excited at the same time, though this does not preclude simultaneous activation or force production in the muscles).

Two modifications are made to our previous model, and they are described here briefly and in more detail in appendix A. First, a passive force term is added to the equations describing force generation within each of the model’s muscles. In other words, each muscle generates force depending on its length *l* and contraction speed $\dot{l}$ according to

2.1 $$f_{\mathrm{tot}}(l,\dot{l},t)=f_{\max}[f_{a}(l,\dot{l})a(t)+f_{p}(l)],$$

where *f*_max_ is the maximum isometric force of the muscle (generated at the optimal muscle length *l*_0_), $f_{a}(l,\dot{l})$ characterizes the muscle’s active force–length–velocity characteristics, *a*(*t*) is the activation state of the muscle at time *t* and *f*_*p*_(*l*) is the length-dependent passive force generated in the muscle. An exponential passive force model (e.g. [20,26]) is used for the present study,

2.2 $$f_{p}(\bar{l})=\left\{ \begin{array}{cc} s_{p}(e^{r_{p}(\bar{l}-l_{p})}-1),\; & \mathrm{if}\;\bar{l}>l_{p}\;\\ 0\; & \mathrm{otherwise},\; \end{array}\right.$$

where $\bar{l}=l/l_{0}$ is the normalized muscle length. This passive force relationship has three parameters: (i) scale *s*_*p*_, (ii) location *l*_*p*_, which is the minimum normalized muscle length at which passive force is generated and (iii) rate constant *r*_*p*_, which determines the exponential growth rate of the passive force when the muscle lengthens above *l*_*p*_.

The second modification made to the model is the addition of an inverse dynamics model in the predictive PD controller which computes muscle excitations. This change enables the controller to predict and counteract unintended movement arising from interaction between connected arm segments (i.e. because segments are kinematically and dynamically coupled via the joints, translation or rotation of one segment causes the connected segments to move as well). Being able to account for these interaction effects helps to maintain dynamic stability^2^ of the arm with a larger range of control parameters which is beneficial when the controller’s ability to compensate for passive stiffness changes is assessed.

### 2.2. Workspace, targets and performance errors

**Figure 1.**
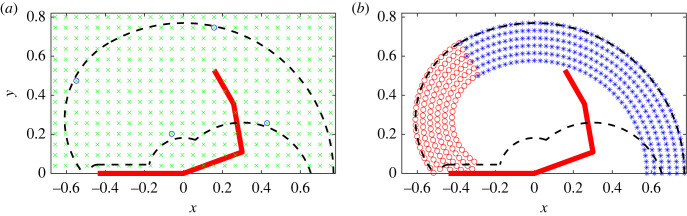
Top-down view of the model’s workspace and target grids with the shoulder at the origin and upper chest represented by the horizontal bar from −0.44 to 0. (*a*) Rectangular target grid (ii) (green crosses), and the four targets of set (i) used for control parameter optimization (blue circles). (*b*) Concentric target grid (iii) near the distal boundaries of the workspace divided into the right (blue asterisks) and left (red circles) boundary sets. Both panels contain the estimated boundaries of the geometrically feasible workspace (dashed black lines) and the initial position of the arm (thick red line).

The approximate boundaries of the model’s geometrical workspace (i.e. all the points in the horizontal plane that the model can reach given its link lengths and the range of motion of the joints) are shown in figure 1. To describe locations or directions within the workspace, we use right/left to refer to ipsilateral/contralateral (as the model comprises right arm only) and proximal/distal to refer to closer/farther from the shoulder joint.

Three sets of targets are used for the simulations (figure 1): the first set (i) is used for adapting the controller to changes in passive stiffness via optimization of the control parameters and the latter two sets (ii–iii) for evaluating the performance of the model with fixed control parameters. Target set (i) is a short, four-target sequence (figure 1*a*), which is suitable for the thousands of simulations required for optimization while still covering the main movement directions within the workspace. Its targets are located near the edges of the workspace, where maximal joint angles are unavoidable, so that parameter optimization is sensitive to passive force changes. Target set (ii) is a rectangular grid of targets (figure 1*a*) which is used to study changes in performance over the entire workspace. Target set (iii) is a grid of concentric targets covering the distal boundary of the workspace and an approximately 20 cm wide band inside it (figure 1*b*), and it is used to investigate changes near the boundary in more detail than possible with the second target set. Target set (iii) is further divided into right and left boundary sets, which correspond to the workspace boundaries drawn by a straight arm over the full range of motion of the shoulder joint (right boundary) and by straight forearm and hand over the full range of motion of the elbow when the shoulder is fully flexed (left boundary). Regardless of the target set, the arm is reset to its initial position (figure 1) between each reach. Unless otherwise stated, targets outside the estimated workspace are included when plotting performance errors over the workspace but omitted from all other analyses.

The performance of the model is measured using two errors to characterize the reach towards each target. The model performs reaching by tracking a pre-planned straight path with a bell-shaped minimum-jerk [27] speed profile to the target, mimicking natural reaching movements (e.g. [28]). The homing-in error *e*_*h*_ measures the distance from the target, averaged over the phase of the movement where, ideally, the endpoint of the arm homes in and stabilizes at the target. This phase lasts from the time the planned trajectory reaches the target to the end of the simulation. Computed this way, *e*_*h*_ serves as a measure of accuracy, but it can also be used to distinguish reaches where the arm is dynamically stable from those where it becomes dynamically unstable. The movement error *e*_mv_ measures how much the arm deviates, on average, from the planned trajectory (i.e. it is the tracking accuracy). In addition to the performance errors, average joint-wise co-activation (i.e. simultaneous non-zero activation state of an antagonistic muscle pair in equation (2.1)) is also computed for each movement. The details of calculating these metrics can be found in appendix A.

### 2.3. Ageing scenarios and parameters

**Figure 2.**
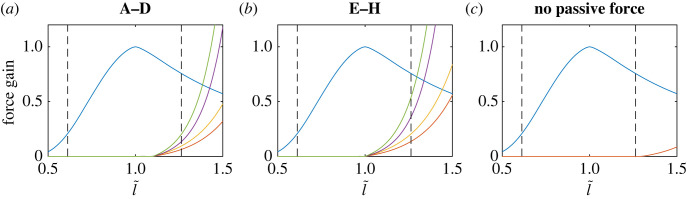
Simulated age-related change in the passive force–length curve. Passive force is plotted against normalized muscle length in the ageing scenarios and case with no passive force (see table 1). For reference, active force–length curve is shown in blue and the length range of the muscles in the model is indicated by vertical dashed lines. (*a*) Cases A (red), B (yellow), C (purple) and D (green). (*b*) Cases E (red), F (yellow), G (purple) and H (green). (*c*) No passive force.

Age-related increase in muscle stiffness does not have a precise definition, but if it is understood as increased passive resistance to elongation at any muscle length, the effect can be achieved using any of the three parameters of the passive force model equation (2.2). Estimating these three parameters from literature is not feasible, however, due to their high sensitivity to experimental noise and uncertainty as well as lack of independence between the parameters near $\bar{l}=l_{p}$. In the present study, we adopt an exploratory approach aimed at capturing a range of realistic passive stiffness increases. In this approach, eight scenarios arising from a low and a high value for each of the three parameters are compared and, for reference, the case with no passive force is also included. A summary of the resulting nine scenarios is given in table 1 with corresponding passive force curves shown in figure 2. The passive force curves are equal for all muscles, but the absolute amount the passive force depends on the muscle-specific isometric strength and the normalized length changes of the muscle during a simulation (see also appendix A). The order of the ageing scenarios, labelled A to H, corresponds to the severity of the passive stiffness effects as measured by the work needed to elongate a muscle to its maximum length in the model. Broadly speaking, the mildest scenario A might be interpreted to correspond to healthy young adults, and hence it will be used as the baseline for comparison, while scenarios B–H represent older adults. The choice of parameter values and their correspondence to experimental data is discussed further below.

**Table 1.** Ageing scenarios and the corresponding passive force parameters. Note that in the no passive force case, muscle length never exceeds *l*_*p*_ so the values of *r*_*p*_ and *s*_*p*_ have no relevance in practice.

scenario	*l*_*p*_	*r*_*p*_	*s*_*p*_
no passive force	1.3	5	0.05
A	1.1	5	0.05
B	1.1	5	0.075
C	1.1	8	0.05
D	1.1	8	0.075
E	1.0	5	0.05
F	1.0	5	0.075
G	1.0	8	0.05
H	1.0	8	0.075

Conceptually, the scaling parameter *s*_*p*_ can be thought to reflect changes in the absolute amount of IMCT. Since the total amount of muscle tissue is constant in the model, *s*_*p*_ also reflects the proportion of IMCT in the muscle. Estimates of age-related increase in the IMCT in humans range from 16% up to 248% [13,15], while ageing rats have been observed to have 39–125% higher area fractions of endomysium and perimysium compared to younger rats [12]. For the present model, we selected *s*_*p*_ = 0.05 as the lower value based on Winters [26] and a 50% increase (resulting in *s*_*p*_ = 0.075) which is conservative but within the range of observed values.

The rate constant *r*_*p*_ grossly represents the material properties of the connective tissue within muscles. Values for parameters comparable to *r*_*p*_ have been reported to increase by 0–45% in rats, depending on the muscle [12,18]. Roughly fitting an exponential function to the passive tension data for muscle fibre bundles from mice [14] and humans [15] suggests an increase in the range of 0–30% in *r*_*p*_. Changes in the optimal muscle length also affect *r*_*p*_, so that, for example, the 14% reduction observed in older mice [29] translates to a reduction of approximately 12% in *r*_*p*_. We chose *r*_*p*_ = 5 based on Winters [26] as the lower parameter value and a relatively high 60% increase to *r*_*p*_ = 8 to balance out the overall low values compared to those used by Thelen [20] to simulate the effects of ageing on ankle function (*r*_*p*_ ≈ 8 for young and *r*_*p*_ = 10 for old muscles).

The location parameter *l*_*p*_ corresponds to the slack length of the IMCT relative to the optimal muscle length. To our knowledge, age-related changes to it have not been directly measured. However, the 10% decrease in the resting fascicle length in humans reported by Narici *et al.* [7] can be taken as an indication of order of magnitude. We choose parameter values based on the relative muscle length excursions observed in the model. At *l*_*p*_ = 1.26, none of the muscles generate passive force in any feasible arm configuration ($\bar{l}<l_{p}$ always), so *l*_*p*_ = 1.3 is used to implement the reference scenario with *f*_*p*_ ≡ 0. On the other end of the scale, at *l*_*p*_ = 1.0 any movement away from the initial joint angles evokes passive force generation from one of the muscles crossing that joint. This value is used for the more severe ageing scenarios, while the baseline scenario is taken to correspond to *l*_*p*_ = 1.1 (9% decrease from young adults to severe stiffening).

The other physical and physiological parameters of the model are set following Murtola & Richards [24]. For the young adult scenario A (baseline) as well as the reference case with no passive muscle force, control parameters (PD gains and prediction time, see appendix A) are obtained by numerical optimization (using mixed-integer genetic algorithm in Matlab, see also appendix A) minimizing the average *e*_*h*_ over target sequence (i). For ageing scenarios B–H, simulations are performed with two sets of control parameters: first with control parameters optimized for the baseline scenario A (called baseline control hereafter) and second with control parameters optimized for the scenario-specific muscle model (called re-tuned control). The re-tuning of the control parameters for scenarios B–H is identical to the optimization of the parameters for the baseline and reference scenarios, but with the known optima for the baseline and reference included in the initial population. The optimized/re-tuned control parameter values can be found in the electronic supplementary material.

## 3. Results

**Figure 3.**
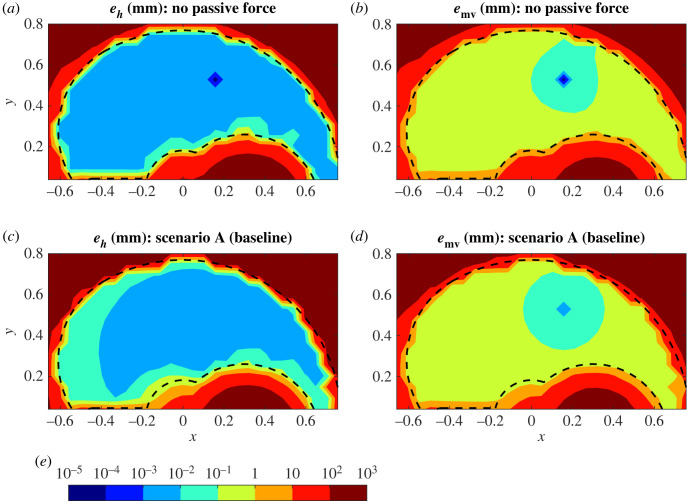
Reaching accuracy for the case with no passive force (*a*,*b*) and the baseline scenario A (*c*,*d*). Homing-in (*a*,*c*) and movement (*b*,*d*) errors are mapped over the workspace using target set (ii). Control parameters have been optimized for each of the scenarios separately. Error contours are log-scaled with colour bar (*e*) values in millimetre and distances are in metres. Dashed black lines indicate the boundaries of the workspace.

Contour maps of the homing-in and movement errors over the model’s workspace are shown in figure 3 for the reference case with no passive muscle force and for the baseline scenario A. Nearly all of the model’s workspace (boundaries in black) can be reached with high accuracy: *e*_*h*_ is typically below 0.1 mm and *e*_mv_ below 1 mm, except for some points closest to the boundaries. The low overall error values in the model can be thought of as the ideal case for human reaching, as the model performs with noise-free signals and with perfect knowledge of the state of the arm and its dynamics. Even under these idealized conditions, the small amount of passive stiffness in scenario A reduces the proportion of the workspace where the highest accuracy reaching can be done (*e*_*h*_ ≤ 0.01 mm), with reaches towards the left (contralateral) distal boundary particularly affected (figure 3*c*). Furthermore, while only one of the targets within the workspace in target set (ii) had *e*_*h*_ exceeding 10 mm when there was no passive muscle force, this increases to 16 targets in scenario A.

**Figure 4.**
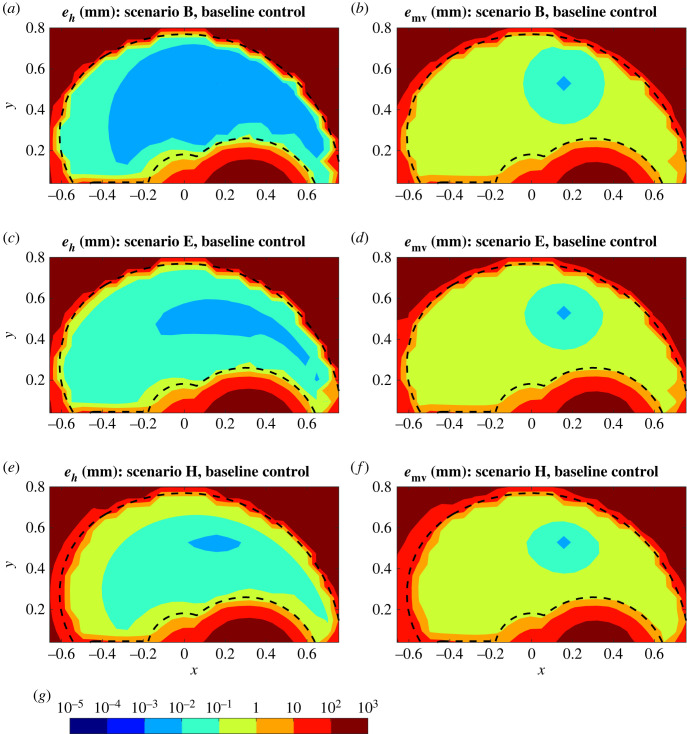
Reaching accuracy for selected ageing scenarios with baseline control. Homing-in (*a*,*c*,*e*) and movement (*b*,*d*,*f*) errors (logarithmic contours, colour bar (*g*) values in mm) are mapped over the workspace (distances in metres) for three ageing scenarios using target set (ii). Scenario B (*a*,*b*) has the mildest and scenario H (*e*–*f*) the most severe ageing effects compared to the baseline case, with scenario E (*c*,*d*) falling in between. Dashed black lines indicate the boundaries of the workspace.

When the passive force generation increases, the global accuracy of reaching suffers without re-tuning of the control parameters (figure 4 for B, E and H, electronic supplementary material for the remaining scenarios). As noted above, the deterioration starts from the edges of the workspace, particularly the distal left (i.e. contralateral) edge which requires maximal shoulder extensor length, and it becomes qualitatively progressively worse with increasing ageing effects. Relative to the baseline scenario A, changes in the performance of the model are relatively small for scenarios B–D (figure 4*a*,*b*), but become more notable for scenarios E–H (figure 4*c*–*f*). This suggests that the global performance of the model over the entire workspace is particularly sensitive to the shift parameter *l*_*p*_. In cases E–H (*l*_*p*_ = 1.0), accuracy is lost near the proximal boundaries of the workspace in addition to the distal boundaries, so that reaching tasks can only be carried out with very high accuracy (*e*_*h*_ ≤ 0.01 mm and *e*_mv_ ≤ 0.1 mm) in the middle of the workspace.

**Figure 5.**
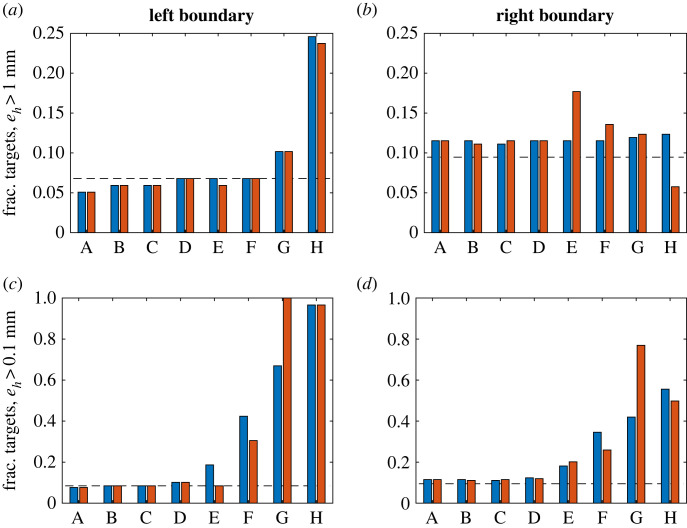
Failure rates at workspace boundaries (target set (iii)). The fraction of boundary set targets (left boundary set in (*a*) and (*c*), right boundary set in (*b*) and (*d*)) where *e*_*h*_ > 1 mm (*a*,*b*) and *e*_*h*_ > 0.1 mm (*c*,*d*) for the eight passive force scenarios in the order of increasingly severe stiffening from A to H (see table 1) with (red) and without (blue) re-tuning of the control parameters. The fraction of failed targets in the no passive force reference case is indicated by the dashed line.

The shrinking of the high-accuracy workspace near the distal boundaries can be quantified using the fraction of targets in the boundary target sets which could not be reached with a given level of accuracy (i.e. *e*_*h*_ over a threshold value). Figure 5 shows the fraction of failed targets for the left and right boundary target set (iii) for two different threshold levels of accuracy, *e*_*h*_ > 1 mm and *e*_*h*_ > 0.1 mm. When the PD control parameters were kept at the baseline values, the failure fraction for the 1 mm threshold case was relatively insensitive to age effects for the left boundary target set, except for the two most severe conditions (G and H; figure 5*a*). By contrast, for the right boundary targets, there was no notable change in the failures with ageing (figure 5*b*). At the higher accuracy requirement level, however, the failure fraction increased for both boundary sets with the severity of passive force generation scenarios, indicating a steadily shrinking high-accuracy workspace (figure 5*c*,*d*).

**Figure 6.**
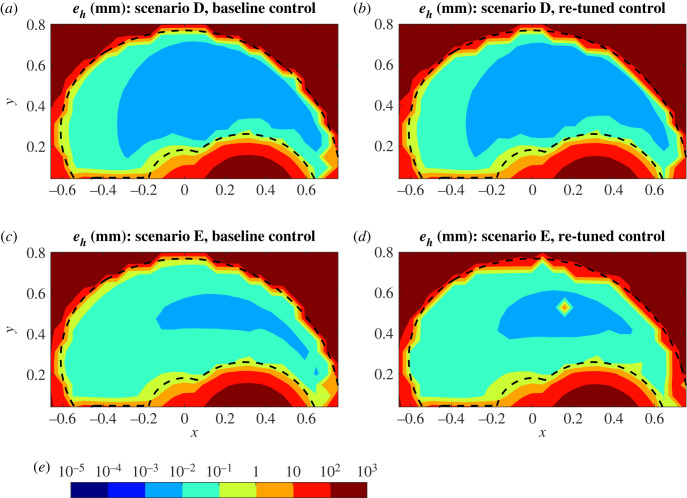
Reaching accuracy before and after re-tuning of control parameters. Homing-in errors mapped over the workspace for scenarios D (*a*,*b*) and E (*c*,*d*) without (*a*,*c*) and with re-tuning (*b*,*d*) using target set (ii). Error contours are log-scaled with colour bar (*e*) values in millimetres and distances are in metres. Dashed black lines indicate the boundaries of the workspace.

The effect of re-tuning of the control parameters in order to improve reaching performance varies from scenario to scenario (figure 6 for D and E, electronic supplementary material for the remaining scenarios). Generally, re-tuning has little impact on the *e*_*h*_ value across the workspace in scenarios B–D (e.g. figure 6*a*,*b*). By contrast, in the more severe ageing scenarios E–H, re-tuning tends to reshape the areas of the workspace with highest reaching accuracy (e.g. figure 6*c*,*d*). This re-shaping can be locally beneficial, for example aligning the area where highest accuracy movements take place with the centre of the visual field. However, in the more severe cases, re-tuning can also be detrimental to global performance, reducing the proportion of the workspace where reasonable performance can be achieved (e.g. *e*_*h*_ < 10 mm). In scenario E, re-tuning also led to lack of stability in the initial arm position (figure 6*d*), which was not observed for any of the other ageing scenarios.

In addition to the baseline control cases, figure 5 also shows the fraction of failed targets in the boundary sets after re-tuning of the control parameters, confirming that the effectiveness of re-tuning varies. The number of failed boundary targets is comparable between baseline and re-tuned control for scenarios A–D, whereas results are typically a mix of increased and decreased failure rates for scenarios E–H.

**Figure 7.**
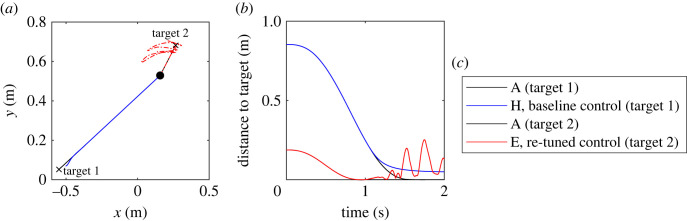
Examples of spatial paths (*a*) and distance to target (*b*) for two different targets. For both targets, reaches are successful in scenario A (black) but stopping-short failure is observed for target 1 for scenario H under baseline control (blue) and oscillatory failure for target 2 for scenario E under re-tuned control (red). The initial position of the endpoint is indicated by a circle and location of the two targets by crosses in (*a*).

The failed reaches, as well as the types of performance deterioration, exhibited by the model fall into two main categories: stopping short and oscillatory/dynamically unstable. Figure 7 illustrates the two failure modes using two targets which are both successfully reached in the baseline case but where success varies in other cases (see also electronic supplementary material, video). In scenario H with baseline control, reaching movement towards the target near the left distal boundary is smooth but stops and stabilizes a short distance from the target (stopping-short failure). In scenario E with re-tuned control, the reach forward and slightly right is failed as the endpoint passes through the target but rather than stopping, the arm starts to oscillate chaotically (oscillatory failure). It is worth noting that not all fails in the second category are chaotic; sometimes the arm simply oscillates with quasi-steady amplitude around the target instead of stopping. The exact targets where oscillatory failure occurred varied between scenarios and control schemes, but the general trend was for oscillatory failures to occur on or near the distal right boundary, while stopping-short type failures tended to occur near the left distal boundary.

**Figure 8.**
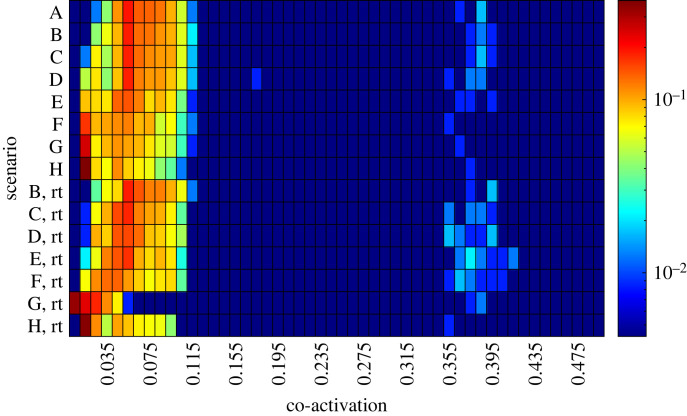
Heatmap of the distribution of co-activation levels across the shoulder in each of the ageing scenarios before (A–H) and after re-tuning (B–H, marked with ‘rt’) in target set (ii). Colour indicates the fraction of targets (logarithmic scale) in the workspace with a given co-activation level.

Co-activation in the present model is an emergent rather than a planned response to inaccuracies in the control signals which make following the planned trajectory challenging [24]. While increasing passive stiffness in the muscles can increase the controller inaccuracies, this does not appear to cause increased co-activation in the simulations. Figure 8 uses the shoulder joint to illustrate the general patterns observed across all three joints (see electronic supplementary material for remaining joints). It uses a heatmap to represent the distribution of the average co-activation level across the workspace (using target set (ii)). For the majority of the workspace, average co-activation remains low for all ageing scenarios (in range 0.01–0.12 for the shoulder, 0.01–0.06 for the elbow and 0.01–0.05 for the wrist), with a few movements requiring a higher co-activation (0.35–0.42 for the shoulder, 0.28–0.37 for the elbow and 0.24–0.33 for the wrist). For comparison, in a typical movement, the average activation level of the muscles remains below 0.2 but can rise as high as 0.8 where there is either a high level of co-activation or a notable level of agonist activation is needed to counteract the passive force from the antagonists in the final position. Regardless of ageing scenario, the higher co-activation level across one or more joints was only observed in movements that were characterized as oscillatory failures, but a small proportion of oscillatory failures occurred without the presence of high co-activation.

## 4. Discussion

We have carried out biomechanical simulations with a simple upper limb model to investigate how age-related increase in passive muscle stiffness affects reaching movements. The simulation results support the hypothesis that as muscles become increasingly stiff, reaching performance starts to deteriorate from the edges of the workspace, progressively shrinking the area where high-accuracy movements can successfully be performed. Contrary to expectations, the simulations did not indicate that re-tuning of the controller would lead to a global improvement in behavioural outcomes, although it could be used to move and reshape the area of the workspace where high accuracy reaching can be carried out.

In simulations with the present model, the deterioration in reaching performance caused by muscle stiffening depends on target location. In particular, performance is largely unaffected for targets close to the location corresponding to optimal muscle lengths (where IMCT is at or below its slack length), while long reaches to contralateral targets are more likely to be affected. The loss of accuracy is also more pronounced on the distal boundaries of the workspace compared to the proximal boundaries, suggesting that the final arm configuration affects reaching outcomes by altering the system’s sensitivity to errors (see [24]) in addition to determining muscle elongations. Experimental data on location specific reaching in the elderly is sparse, but some evidence of similar patterns exists. In long reaches, target location has been observed to affect accuracy more in older than in young adults; in particular, reaches using large shoulder flexion resulted in loss of accuracy in the elderly [30]. By contrast, other experiments used shorter reaches to look at the primary submovement (i.e. the initial movement to the general vicinity of the target, prior to homing in with secondary submovements). The results suggest no age–location interaction effect on the proportion of the distance to target covered by the primary submovement [31]; in other words, the loss of initial accuracy before corrections did not appear to be sensitive to target location in shorter movements. Further experiments are needed, however, to understand whether the age-related performance deterioration highlighted by our simulations is also observed in multi-dimensional reaching across the entire workspace by the elderly.

Multiple studies (e.g. [32–34]) have noted that in comparison to younger adults, older adults use shorter primary submovements and more corrective secondary movements, that is, they use the so-called play-it-safe strategy. The present model plans each reach as a single, primary submovement followed by *ad hoc* corrections which, when successful, produce exponential convergence towards the target. Hence, the model’s ability to replicate the play-it-safe strategy with multiple distinct submovements is limited without the addition of continuous or intermittent planning (such as [35,36]). However, the stopping-short failure mode seen in our simulations (figure 7) suggests that age-related increase in passive stiffness may cause the primary submovement to become shorter if active agonist contractions are not increased sufficiently to overcome passive antagonist forces. Furthermore, if the relatively unalterable passive muscle stiffening contributes to the reliance on secondary submovements by the elderly, it would help to explain why practise has been observed to have relatively little effect on the submovement structure in older adults [32] even though overall reaching performance improves at comparable rates in both older and young participants [32,37,38].

Another well documented characteristic of reaching by the elderly is slowness, and in most experimental settings, both young and older subjects have been observed to achieve comparable accuracy as long as they are allowed to adjust their speed of movement (e.g. [34,37,39]). When required to move at a matched speed with a younger group, older adults may lose final endpoint accuracy [39] or need to make more corrective submovements [40]. A variety of factors behind the age-related slowing of movements has been suggested, including reduced ability of muscles to generate the force needed for faster movements, alterations in planning or visual information processing, and changes in preferred movement strategy (see [41]). As the aim of the present study was to investigate the effect of muscle stiffness in isolation, these other age-related changes were excluded from the model, and hence, movement speed was outside the current scope. It is also worth noting that altering the speed of the movement cannot help to overcome the stopping-short type failures, because they arise from static force balances.

Even though the causes of slowness are outside the current scope, omitting it as a compensation strategy may have implications for the controllability of the present model. In particular, slower movement speed might counteract, to some extent, the destabilizing effects of increasing the PD gains of the controller, as smaller changes to the control signals would be needed. Thus, combining slower movements with re-tuning might allow the controller to improve performance via higher PD gains without simultaneously increasing oscillatory failures, and hence this might form a successful strategy for dealing with increasing passive stiffness. The potential benefits of higher PD gains are also suggested by the fact that the baseline gains were an order of magnitude higher than those for the reference case with no passive force at all, and this increase in the gains allowed the model to retain good performance over most of the workspace. Higher controller gains can be interpreted as increased emphasis on following the planned trajectory exactly, which can in some circumstances lead to beneficial outcomes for the elderly (e.g. the straighter path to one of the four targets used by Seidler *et al.* [30]) but when combined with reduced ability to execute the movements, the result can be increased jerkiness of movement, as often observed in older test subjects (e.g. [30,31]). It is worth noting that the constant control parameters used for all targets represent a minimalist control scheme, and, further, the parameter values are obtained looking at performance on a limited set of targets. Optimizing or re-tuning parameters using a different or more comprehensive set of targets might result in performance improvement for some regions of the workspace, but given the limited number of control parameters (seven) and the infinite possible target placements within the workspace, it is unlikely that a different re-tuning procedure would completely remove age-related effects on reaching performance. A more complex control strategy involving location or direction specific gains or gains varying throughout the movement has potential to resolve some of the issues but comes at a significantly higher cost for obtaining and storing such gains.

It has been hypothesized that one of the major factors behind age-related changes in motor performance is a reduced ability to plan movements (e.g. [42]). While evidence suggests that this deficiency may not affect maximal-speed single-joint movements [41], it remains a viable explanation for performance deterioration or control adaptations for complex, multi-joint movements, such as three-dimensional reaching against gravity [43] and tasks requiring compensation for the variable joint torques arising from interactions between arm segments [44]. In the present model, motor planning relies on virtually perfect forward and inverse models of the arm, which correspond to internal models in the human neuromuscular control system (see e.g. [45]). These internal models have been suggested to change with age [46] or to fail to be updated with age-related changes [47]. In the present model, such reduced-accuracy internal models would likely result in further performance deterioration as correcting movement errors introduced by the mismatch between the internal models and the dynamic behaviour of the arm increases the likelihood of oscillations. This increased tendency for oscillations also reduces the effectiveness of re-tuning, emphasizing further that low-level controller adaption to ageing, represented by re-tuning of control parameters, is unlikely to be an efficient strategy to universally improve performance.

Co-contraction (i.e. simultaneous force production) of antagonistic muscle pairs, which effectively increases joint stiffness, has been proposed as a means of controlling movement accuracy, particularly in the presence of uncertainties (see e.g. [45]). Experimental studies have also suggested that when muscle activity is measured using surface electromyography (EMG), ‘co-EMG’ may increase with age during tasks such as maximal voluntary contractions [48] and walking on level ground [49] and on stairs [50]. Our model does not contain a variable that corresponds directly to surface EMG, but qualitative comparisons can be made with the co-activation level computed for each joint. The control strategy used in the present model precludes co-contraction arising from co-excitation, that is, as a high-level planned movement strategy. However, the model exhibits automatic co-activation caused by the activation dynamics of the muscles when desired joint torques change direction, and our simulation results suggest that this mode of co-activation does not vary with passive muscle stiffness. By considering the net joint torque arising from co-activation, it can also be concluded that co-activation, whether automatic or planned, cannot counteract performance deterioration or failures of stopping short type except in arm configurations where the isometric strength and force–length gains favour the agonist significantly. Hence, the model behaviour suggests that age-related changes in co-contraction are more likely to be related to other age-related changes in the neuromuscular system than to compensation for increased passive muscle stiffness. Implementing a control strategy which allows co-excitation and is hence suitable for testing this hypothesis is left for future work.

One major way in which the model behaviour does not align with observations from humans is the presence of oscillatory failures. These oscillations were observed for all scenarios with passive stiffness enabled (A–H) under both baseline and re-tuned control, but not for the reference case with no passive muscle force. Additional simulations for scenarios A–H using the control parameters optimized for the reference case (not reported herein) suggest that the presence of oscillations is not due to the increased passive stiffness *per se*, but because counteracting the deteriorating effects of this stiffening requires higher PD control gains and a shorter prediction time. While higher controller gains allow for larger correctional torques, and hence improve accuracy, they are also a known source of instability in systems under PID control [51]. Similarly, the shorter prediction time reduces the errors inherent in predicting the system state, but it also reduces the controller’s ability to account for delays in muscle contraction. This can potentially leave significant delays in the control loop, which may cause instability [52]. The presence of these oscillations in the model when none are observed in humans suggests that the model lacks at least one key stabilization mechanism. As discussed above, this mechanism could be the ability to alter movement speed, the use of gains that vary with task, arm configuration or time, or the ability to increase co-contraction via co-excitation. It is also possible that humans use alternative strategies, such as moving the whole body, which limit the need to maintain high performance across the entire reaching workspace. Furthermore, as the controller has not been constructed to be an accurate representation of a neural motor control system, it is possible that instability may arise from over-reliance on PD-type feedback where human motor control uses more sophisticated strategies.

### 4.1. Limitations

A central limitation of our study is the modelling of passive stiffness to mimic the ageing process. Due to ambiguities in passive stiffness data in literature, we were unable to pinpoint whether the ‘stiffening’ of ageing muscles stems from a simple leftward shift, increased scaling or shape changes of the passive force–length curve. Thus, we attempted to parameterize the passive force curve to capture all possible features from the experimental data. We further note that although the standard passive force model, equation (2.2), is commonly used, alternative models have also been proposed (e.g. [17,53]). Furthermore, the choice of parameters in the exponential passive force models is also ambiguous, with many studies assuming *l*_*p*_ = 1 (e.g. in [20,26,54]) even though estimation of *l*_*p*_ from experimental data suggests that it plays a role in musculoskeletal changes, for example, after a stroke [55]. The question of how to ideally model the properties of IMCT is outside the scope of the current work, but changes in *l*_*p*_ effectively enable or disable other passive stiffening mechanisms to affect reaching performance in the present model. Our results hence highlight the need for better understanding of the passive properties of muscle tissue and how they might change in response to age, injury or disease. This in turn requires high-quality data, particularly near the optimal and slack fibre lengths, so that challenging-to-infer parameters such as *l*_*p*_ can be studied.

There is also a scarcity of available data on age-related passive stiffening in the human upper limb. Studies in rodents suggest that muscle stiffening may not happen uniformly across all muscles [12], and it has also been observed that the severity of other age-related changes, such as loss of muscle strength and volume, vary between human upper limb muscle groups [6]. However, in the absence of further information about muscle stiffening in human upper limbs, the present model relies on identical ageing changes in all muscles, and hence it may under- or overestimate the effects on the movement of individual joints. However, as performance deterioration in the model was mainly sensitive to stiffening of the shoulder muscles which undergo large relative length changes, the main patterns observed in simulations are unlikely to disappear if the relative stiffening of elbow or wrist muscles is altered.

The modelling approach chosen for the current study also comes with its own set of limitations. As passive muscle stiffening was studied in isolation, interaction effects with other age-related musculoskeletal or neural changes were excluded. Such interaction effects could include, for example, an increase of passive stiffness in relative rather than absolute terms, which has been suggested based on the observation that active contractions become weaker with age while passive muscle properties remain unchanged [56]. Moreover, the increase in passive force could occur with, and be partially caused by, age-related reduction in optimal muscle lengths [29]. Tendons, which affect force transmission and a muscle’s length and velocity trajectory during movements, may also undergo age-related structural changes [57], although the changes in tendon stiffness can be insignificant in the upper limbs [58]. Due to the nonlinearity of the arm model, the consequences of multiple such changes are hard to predict, and hence remain a subject for further study. Furthermore, an anatomically more realistic three-dimensional model is needed to infer where real muscles operate in their passive force curves during real reaching tasks, as well as to ensure that simulations are more easily compared with experimental data.

One of the fundamental factors affecting the behaviour of any musculoskeletal model is the muscle model used. In the present model, we use a Hill-type model with no tendons. Like Hill-type models in general, the current model represents the steady-state characteristics of muscle contractions but omits transient effects such as short-range stiffness [59] which contributes to the muscle perturbation response. Elastic behaviour of muscle fibres, typically represented by a series elastic component separate from tendons (e.g. [60]), is also omitted. As our reaching movements were dynamic and relatively slow, muscle behaviour is expected to be dominated by the steady-state force–velocity–length-activation characteristics [61], but the absence of short-range stiffness could contribute to the observed instability in the model. Hence, future work should investigate how transient and dynamic muscle characteristics modulate the observed reaching behaviour. The augmentation of the muscle model to include transient effects (e.g. [62]) is particularly important if the tasks for the present model are expanded to include, for example, short, fast movements or perturbations.

The omission of tendons in the model has an impact on the dynamical behaviour of the system, as tendon lengthening and shortening will affect the length–velocity operating point of muscles in a force-dependent manner. For the present model and task set, the impact is expected to be relatively small, as positional tendons, such as those in the arm, typically only experience length changes of approximately 2–3% in use [63]. Furthermore, any change in a muscle’s operating point is automatically accounted for by the controller, enabling muscles to generate the desired force to execute each movement regardless of the presence or absence of tendon dynamics as long as movements remain relatively slow. As with muscles, age-related changes in upper limb tendons are under-researched, so it is possible that simultaneous changes in tendon and IMCT properties could either enhance or mitigate some of our results. Investigating this is, however, outside the scope of the present work.

### 4.2. Summary and conclusion

Despite the limitations of the present approach, its simplicity enables thorough exploration of the model’s parameter space to investigate the effects of muscle stiffening on reaching performance. From our simulations of rapid goal-directed reaching, our central findings are as follows. (i) Reaching performance is sensitive to the passive stiffness of muscle; as the severity of stiffening increased, the area of reachable workspace diminished. (ii) Re-tuning of the neural controller cannot fully reverse the effects of muscle stiffening, but it can move and reshape the reachable workspace. (iii) Among all simulations, two failure modes were observed due to age-related stiffness. In one mode, the model stopped short of the target which is reminiscent of the play-it-safe strategy observed in the elderly (e.g. [39,41]). In the other mode, the controller failed to stabilize the arm at the target, causing oscillations. (iv) In all simulated ageing scenarios, failure and performance deterioration was most likely to occur for far reaches (e.g. targets on the contralateral side and on the distal boundaries of the reachable workspace).

The above findings of the present study are evidence that the passive mechanical properties of muscle can impact neuromuscular control of goal-directed movements. Future computational studies will be required to further investigate how more realistic musculoskeletal anatomical features impact the effects of age-related stiffening on reaching. Additionally, further experimentation is required to more precisely measure passive force–length properties so that the effects of ageing can be more accurately parameterized. Finally, future experimental and computational work is required to investigate how adaptation of behavioural, planning or control strategies might be used to mitigate the effects of age-related alterations of muscle properties.

## Figures and Tables

**Figure 9.**
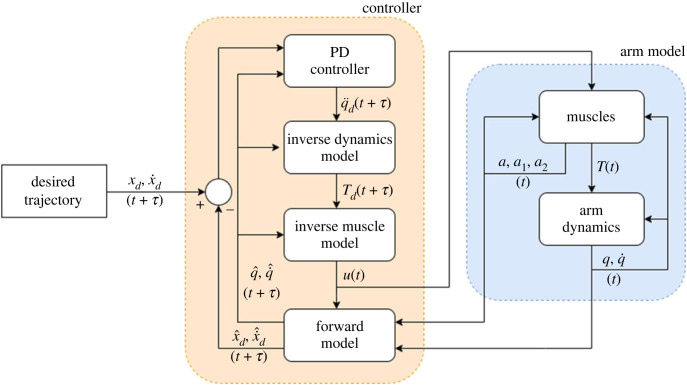
The control scheme of Murtola & Richards [24] has been updated with an inverse dynamics model to compute the desired torques.

## Data Availability

Data and relevant code for this research work are stored in GitHub: https://github.com/tmmurtola/reaching-arm-model and have been archived within the Zenodo repository: https://doi.org/10.5281/zenodo.7545405 [64]. The data are provided in the electronic supplementary material [65].

## Footnotes

1 For convenience, we call horizontal adduction/abduction of the shoulder joint flexion/extension as well.

2 Here understood as the ability of the system to follow a desired trajectory and to return to it after a disturbance.

## Appendix A

**A.1. Muscle model**

To expand equation (2.1) more formally, the force generated by muscle *j*, *j* = 1, 2, …, 6 in the model is

A 1 $$f_{\mathrm{tot},j}({\bar{l}}_{j},{\bar{v}}_{j},t)=f_{\max,j}[f_{a}({\bar{l}}_{j},{\bar{v}}_{j})a(u_{j}(t))+f_{p}({\bar{l}}_{j})],$$

where *f*_max,*j*_ is the constant isometric strength of the muscle. The active force gain of the muscle is determined by its force–length–velocity characteristics at normalized muscle length ${\bar{l}}_{j}=l_{j}/l_{0,j}$ and speed ${\bar{v}}_{j}={\dot{l}}_{j}/l_{0,j}/v_{\max}$, where *v*_max_ is the maximum contraction speed,

A 2 $$f_{a}({\bar{l}}_{j},{\bar{v}}_{j})=f_{l}({\bar{l}}_{j})f_{v}({\bar{v}}_{j}),$$

where the force–length curve is

A 3 $$f_{l}(\bar{l})=e^{-|({\bar{l}}^{b_{2}}-1)/b_{3}{|}^{b_{1}}},$$

with shape parameters **b** = (*b*_1_, *b*_2_, *b*_3_), and the force–velocity curve is

A 4 $$f_{v}(\bar{v})=\left\{ \begin{array}{cc} \frac{1-\bar{v}}{1+d_{1}\bar{v}},\; & \mathrm{if}\;\bar{v}>0\;\\ d_{2}-\frac{\left(d_{2}-1)(1+\bar{v}\right)}{1-d_{3}\bar{v}},\; & \mathrm{otherwise}\; \end{array}\right.$$

with shape parameters **d** = (*d*_1_, *d*_2_, *d*_3_). In addition to the force–length–velocity characteristics of the muscle, the active force generated also depends on the activation state *a*(*u*_*j*_(*t*)) ∈ [0, 1] which describes the temporal response of the muscle to excitation *u*_*j*_(*t*). The third-order model [25] is used for the excitation to activation mapping *a*. All muscles are assumed to have identical force–length–velocity characteristics and activation dynamics (see [24] for parameter values and further details), but they differ in their isometric strengths *f*_max,*j*_ and optimal lengths *l*_0,*j*_, as well as the state of the muscle at a point in time in the simulation $\left({\bar{l}}_{j},{\bar{v}}_{j},u_{j}\right)$.

**A.2. Controller**

The predictive PD controller of Murtola & Richards [24] is modified to include a model for inverse dynamics (figure 9). As in the original controller, the desired trajectory, described by two-dimensional endpoint position **x**_*d*_(*t*) and velocity ${\dot{x}}_{d}(t)$, is a straight line with a bell-shaped minimum-jerk [27] speed profile, and reaching is framed as a trajectory tracking task rather than trajectory optimization task. The desired torques **T**_*d*_, used to compute muscle excitations, *u*_*j*_, are computed in two steps. First, the forward model predicts the state of the system at time *t* + *τ*, where *τ* is the prediction time, which the PD controller uses to calculate desired joint accelerations ${\ddot{q}}_{d}={\ddot{q}}_{d}(t+\tau )$

A 5 $${\ddot{q}}_{d}=K_{p}J^{†}(\hat{q})(x_{d}-\hat{x})+K_{v}J^{†}(\hat{q})({\dot{x}}_{d}-\hat{\dot{x}}),$$

where **K**_*p*_ and **K**_*v*_ are the diagonal gain matrices, $J^{†}(\hat{q})$ is the pseudo-inverse of the Jacobian at the predicted joint angles $\hat{q}$ and $\hat{x}$ and $\hat{\dot{x}}$ are the predicted endpoint position and velocity, respectively.

In the second step, the desired torques are computed to account for the model’s dynamics, except for contact forces, by

A 6 $$T_{d}=M(\hat{q}){\ddot{q}}_{d}+c(\hat{q},\hat{\dot{q}}),$$

where $M(\hat{q})$ is the predicted inertia matrix of the arm and $c(\hat{q},\hat{\dot{q}})$ is the vector containing predicted Coriolis and centrifugal effects. The computation of *u*_*j*_(*t*) for the current time step from **T**_*d*_(*t* + *τ*) produced by equation (A6) is identical to the original model, effectively assigning flexion torques to flexor muscles and extension torques to extensor muscles (i.e. assuming no co-excitation of the antagonist muscle pairs), using the muscle moment arms computed in the course of the simulation and equation (A1) to find *a*_*j*_(*t* + *τ*) and then using a constant delay approximation of the activation dynamics to carry out the inverse mapping $a_{j}(t+\tau )⟶u_{j}(t)$.

**A.3. Performance measures**

The homing-in error is the average deviation of the two-dimensional endpoint position **x**(*t*) = (*x*(*t*), *y*(*t*)) from the target location (denoted **x**_*t*_) after the planned endpoint speed reaches zero (at time *T*_*p*_),

A 7 $$e_{h}=\frac{1}{T_{\max}-T_{p}}\int_{T_{p}}^{T_{\max}}\|x_{t}-x(t)\|\;dt,$$

where *T*_max_ is the duration of the simulation. We use *T*_max_ = 4 s, which ensures that *e*_*h*_ is dominated by the final behaviour of the model, whether that is a convergence to a stationary position (*e*_*h*_ ≈ final accuracy), quasi-stable oscillations around **x**_*t*_ (*e*_*h*_ ≈ average accuracy), or divergent/chaotic movement (*e*_*h*_ large compared to the two other behaviours). Note that *e*_*h*_ closely resembles the stabilization error used by Murtola & Richards [24].

The movement error characterizes the deviation of the endpoint from the planned trajectory throughout the simulation,

A 8 $$e_{\mathrm{mv}}=\frac{1}{T_{\max}}\int_{0}^{T_{\max}}\|x_{d}(t)-x(t)\|\;dt.$$

Average co-activation *γ*_*i*_ for joint *i* = 1, 2, 3 is computed using the activation states (see equation (A 1)) of the two muscles crossing that joint. If these muscles are indexed, say, 2*i* and 2*i* − 1, then

A 9 $$\gamma_{i}=\frac{1}{T_{\max}}\int_{0}^{T_{\max}}\min(a(u_{2i}(t)),a(u_{2i-1}(t)))\;dt,$$

where the min ( · , · ) function selects the smaller of its two arguments at any point in time. Note that even though the control strategy precludes simultaneous excitation of antagonistic muscles (i.e. if *u*_2*i*_(*t*) > 0 then *u*_2*i*−1_(*t*) = 0 and vice versa), the model can still produce significant levels of co-activation because the activation state takes time to develop and decay after changes in excitation.

Control parameters **K**_*p*_, **K**_*v*_ and *τ* are optimized numerically using mixed-integer genetic algorithm in Matlab (*τ* is discrete as it corresponds to timesteps in simulations). The objective is to minimize

A 10 $${\tilde{e}}_{h}=\frac{1}{4}\sum_{\;p=1}^{4}\tanh\left(\frac{e_{\mathrm{st},p}}{\epsilon_{\mathrm{tol}}}\right),$$

where the homing-in error for the *p*th target in the four-target sequence is denoted *e*_*h*,*p*_. The tanh-transformation together with the tolerance $\epsilon_{\mathrm{tol}}=1\;\mathrm{cm}$ ensures that if one of the targets is functionally unreachable, it does not dominate the optimization.
